# Perceived social support partially mediates the association between childhood abuse and pain-related characteristics

**DOI:** 10.3389/fpain.2022.1075605

**Published:** 2022-12-22

**Authors:** Jennifer Pierce, Jacob Presto, Elizabeth Hinckley, Afton L. Hassett, Joseph Dickens, Jill R. Schneiderhan, Kathryn Grace, Jenna McAfee

**Affiliations:** ^1^ Department of Anesthesiology, University of Michigan, Ann Arbor, MI, United States; ^2^ Department of Pediatric Neurology, Vanderbilt University Medical Center, Nashville, TN, United States; ^3^ Department of Family Medicine, University of Michigan, Ann Arbor, MI, United States.

**Keywords:** childhood abuse, emotional support, instrumental support, widespread pain, pain severity, depressive symptoms, anxiety symptoms, social support

## Abstract

Higher perceived social support has been shown to buffer the impact of negative stressful events like childhood abuse on health outcomes. Yet, the role of perceived social support as a mediator of the association between childhood abuse and pain-related characteristics is not well understood. The present study explored this premise. Patients (*n* = 1,542) presenting to a tertiary-care, outpatient pain clinic completed a cross-sectional survey consisting of regularly collected clinical data and validated measures. Path analysis suggested that the impact of childhood abuse on sensory and affective pain-related characteristics was partially explained by perceived emotional support. Survivors of childhood abuse display a more complex clinical pain phenotype and this extends to more negative perceptions of social support. Our findings may reflect processes whereby childhood abuse negatively impacts social relationships across the lifespan, and these negative social perceptions and relationships influence sensory and affective components of pain.

## Introduction

Trauma exposure and chronic pain are strongly associated (1). Childhood abuse, in particular, has been linked to the prevalence of chronic pain and pain-related characteristics. A history of childhood maltreatment is associated with more physical symptoms, a higher likelihood of a number of pain conditions (e.g., back pain; headaches), greater anxiety and depressive symptoms in adulthood, and a more complex clinical phenotype among chronic pain patients (2–5). Investigating mechanisms of the association between childhood abuse and pain-related characteristics is imperative to understanding and addressing this complexity. Indeed, numerous factors have been proposed as mechanisms driving the mutual maintenance of trauma and pain symptoms, including attentional biases toward threat or pain-related stimuli and expectations of negative outcomes (1). In the present study, we propose that the association between childhood abuse and chronic pain may be partially explained by perceptions of social support, or the belief that others are available to provide help and care (6).

Childhood abuse may be particularly damaging to perceptions of social support by altering the way in which individuals perceive and relate to others (7, 8). According to Miller et al. (9), childhood abuse is associated with negative behavioral tendencies including threat vigilance and mistrust of others, which can make social relationships in adulthood more tenuous and have downstream effects on physical health (8, 9). This extends to lower perceptions of social support, which have been linked to a history of childhood maltreatment (10–13).

Perceived social support is an important predictor of health and well-being in general (14) and in the development and maintenance of chronic pain in particular (15, 16). Previous research suggests that higher perceived social support buffers the impact of negative stressful events on health outcomes, including pain-related and psychological outcomes (14, 17). Yet, research also suggests a direct association between lower social support and pain-related characteristics, including higher pain severity, higher pain sensitivity, and higher negative affect (17–22). Mechanistically, higher perceived social support is associated with lower levels of inflammation (23), whereas chronic stress resulting from negative relationship experiences may lead to physiological dysregulation (24). In turn, neuroendocrine and inflammatory biomarkers are hypothesized to play a role in chronic pain for some patients (25, 26).

Interestingly, few studies have considered social support as a potential mechanistic pathway linking childhood maltreatment to physical and mental health in adulthood despite evidence that childhood abuse impacts social perceptions. Some studies provide evidence of this pathway (12, 13, 27) while others do not (28). To our knowledge, only one study has evaluated social perceptions in the association between childhood abuse and pain-related characteristics. Alhalal et al. (27) found that, in a sample of married Saudi women, perceptions of family support mediated the association between childhood abuse and depressive symptoms, which then exhibited a direct association with chronic pain severity (i.e., pain intensity and disability).

Despite finding support for this potentially important association, their study was limited by a narrow conception of social support and pain-related outcomes. We sought to advance this research in three ways. First, we assessed these associations in a sample of patients with pain. Second, we evaluated both perceived emotional (perception of love and caring from others) and perceived instrumental social support (perception of tangible help being available when needed). Third, because pain is a multifaceted experience (15, 16), we considered both sensory (i.e., widespread pain and pain severity) and affective (i.e., anxiety and depressive symptoms) components as outcomes. We sought to determine: (a) whether childhood abuse was associated with perceived emotional and instrumental social support, as well as widespread pain, pain severity, anxiety symptoms and depressive symptoms; (b) whether perceived emotional and instrumental social support were associated with widespread pain, pain severity, anxiety symptoms, and depressive symptoms; and (c) whether perceived emotional and instrumental social support partially mediated the association between childhood abuse and the aforementioned pain-related characteristics. We hypothesized that the impact of childhood abuse on widespread pain, pain severity, anxiety symptoms, and depressive symptoms would be partially mediated through perceived emotional and instrumental social support.

## Methods

### Participants and procedure

Patients 18 years of age and older presenting for care at the University of Michigan Back & Pain Center between June 2018 and March 2020 were included in the present analyses. The Back & Pain Center is a tertiary-care, outpatient clinic servicing chronic pain patients across a range of conditions. All new patients are provided with a packet of questionnaires to complete prior to their first visit to be used for clinical care and research purposes (29). University of Michigan Medical School Institutional Review Board (HUM00041820) approval was obtained. A waiver of informed consent was obtained due to the extensive use of the survey in a clinical setting. Remuneration was not provided. Prior studies have utilized the patient data obtained through the Back & Pain Center; however, our specific sample and research question are unique compared to these previous studies (4, 30).

### Measures

#### Demographics

Age, sex, and marital status were obtained.

#### Abuse history

Patients were asked three questions assessing abuse history: *Do you have a history of psychological or emotional abuse?*; *Do you have a history of physical abuse?*; and *Do you have a history of sexual abuse?* Response options included 1 (Yes) and 0 (No). Patients who responded yes were asked to indicate when the abuse occurred, including childhood (<13 years), adolescence (13–18 years), and adulthood. For the present study, *childhood abuse* included experiences in childhood and adolescence. We combined psychological or emotional, physical, and sexual abuse into a single measure of abuse for multiple reasons. First, our central question focused on social support as a mediator, rather than potential differentiation of types of abuse. Second, different types of abuse frequently co-occur. In the present data, 85% of individuals who self-reported physical abuse also reported emotional abuse and 47% reported sexual abuse. Similarly, 66% of individuals reporting sexual abuse also reported emotional abuse. Conversely, of those who did not report emotional abuse, only 1.5% reported physical abuse and 3.5% reported sexual abuse. Third, this is a simpler way for clinicians to assess heightened risk rather than evaluating specific types of abuse experienced by patients, making the findings more clinically useful. Therefore, if a participant indicated a history of any form of abuse during childhood or adolescence, they were considered to have had a history of childhood abuse.

#### Widespread pain

Widespread pain was assessed with the Michigan Body Map (31), which included a sum of 35 potential body areas. Participants were asked to mark the areas of the body where they felt persistent or recurrent pain present for the last three months or longer. Scores ranged from 0 to 35 (31–33).

#### Pain severity

The 4-item Brief Pain Inventory was used to assess pain severity (34). Patients were asked to rate their pain at its worst in the last week, least in the last week, on the average, and right now, with options ranging from 0 (no pain) to 10 (pain as bad as you can imagine). An average score was calculated.

#### Anxiety and depressive symptoms

Anxiety and depressive symptoms were assessed using the Patient Reported Outcomes Measurement Information System (PROMIS) 8-item scales (Anxiety-Short Form 8 and Depression-Short Form 8a, respectively). Patients were asked to consider symptoms in the past seven days (e.g., *I felt fearful* [anxiety symptoms]; *I felt worthless* [depressive symptoms]). Response options ranged from 1 (never) to 5 (always). Sum scores were obtained for each scale and ranged from 8 to 40.

#### Perceptions of social support

Perceptions of emotional and instrumental support were assessed with the PROMIS four-item social support scales. Perceived emotional support was assessed using the PROMIS Item Bank v2.0—Emotional Support—Short Form 4a (e.g., *I have someone who will listen to me when I need to talk*). Perceived instrumental support was assessed using the PROMIS Item Bank v2.0—Instrumental Support—Short Form 4a (e.g., *Do you have someone to help you if you are confined to bed?*). For both measures, response options ranged from 1 (Never) to 5 (Always). Scores are summed for each scale and range from 4 to 20.

### Data analysis plan

Patients were excluded if they did not provide information regarding abuse history. Additionally, if a participant indicated “no” to one or two forms of abuse, but left any missing, they were treated as missing in the final analyses. Sex, marital status, and childhood abuse were dummy coded, such that male sex, being nonmarried, and no childhood abuse were reference categories. Descriptive statistics were obtained for the variables of interest. Differences in the variables of interest by history of childhood abuse were assessed using *t*-tests and contingency analyses with associated *χ*^2^ tests. Bivariate correlations were also assessed. Variance inflation factor did not exceed 3.0 for any variable; thus, multicollinearity was not a concern for the path model.

Path analysis was used to assess whether perceived emotional and instrumental support mediated the association between childhood abuse and widespread pain, pain severity, and negative affect. Mediators were allowed to covary as were the outcome variables. Childhood abuse was allowed to associate with outcomes conditional on the mediators. Thus, the model evaluated the presence of partial mediation using a saturated model and model fit statistics were not obtained. Sex, age, and marital status were included as covariates for each mediator and outcome. Full information maximum likelihood method of estimation and robust standard errors were used to account for missing values and non-normal data. Squared multiple correlation coefficients (R^2^) were used to determine the variance explained in each dependent variable.

A bootstrap procedure with 10,000 replicates was used to create bias-corrected and accelerated (BCa) 95% confidence intervals (95% CI) for the indirect effects. Complete case analysis was used to evaluate the significance of the indirect effects. Confidence intervals not including zero were considered significant. Total indirect effect coefficients were divided by total effect coefficients for each outcome to determine how much of the total effect was accounted for by the indirect effects through perceived emotional and instrumental support. Additionally, specific indirect path coefficients were divided by the total indirect effect coefficients to determine the relative importance of each pathway. The sum of the absolute values was used for the total indirect effect. Analyses were run using Stata version 15 (College Station, TX).

## Results

The full sample consisted of 1,542 patients (*M*_age_ = 54.0, *SD* = 16.4; 54.9% female). Approximately 19.5% (*n* = 300) reported a history of childhood abuse. Individuals who reported a history of childhood abuse were younger, more often female, and less often married. Additionally, compared to individuals who reported no history of childhood abuse, those who reported childhood abuse exhibited lower perceived emotional support, lower perceived instrumental support, more widespread pain, higher pain severity, higher anxiety symptoms, and higher depressive symptoms. See Table 1 for descriptive statistics and comparisons by history of childhood abuse.

**Table 1:** Descriptive statistics and group differences by history of childhood abuse.

			History of childhood abuse		
	Total		No	Yes	
	(*n *= 1,542)	Range	(*n *= 1,242)	(*n* = 300)	*p*
Age	54.0 (16.4)	18–98	54.8 (16.7)	50.7 (14.7)	<.001
Female, % (*n*)	54.9% (847)	–	51.5% (640)	69.0% (207)	<.001
Married, % (*n*)	57.6% (844)	–	60.1% (709)	47.2% (135)	<.001
Perceived emotional support	17.4 (3.6)	4–20	17.7 (3.4)	16.4 (4.0)	<.001
Perceived instrumental support	17.1 (3.9)	4–20	17.4 (3.7)	15.8 (4.5)	<.001
Widespread pain	7.5 (5.9)	0–35	7.0 (5.6)	9.5 (6.8)	<.001
Pain severity	5.9 (2.0)	0–10	5.8 (2.0)	6.2 (1.7)	.002
Anxiety symptoms	17.6 (7.9)	8–40	16.7 (7.5)	21.5 (8.3)	<.001
Depressive symptoms	15.4 (7.9)	8–40	14.5 (7.3)	18.9 (8.9)	<.001

Values are mean (SD) unless otherwise noted. Sample sizes vary due to missingness. Omnibus *χ*^2^ tests were conducted for categorical variables and independent samples *t*-tests were conducted for continuous variables. Missing values were not included in the contingency analyses and associated *χ*^2^ tests. Percentages for contingency analyses are within column.

Perceived emotional and instrumental support were significantly positively correlated. Higher perceived emotional support and perceived instrumental support were also significantly associated with less widespread pain, lower anxiety symptoms, and lower depressive symptoms. All of the model outcomes were significantly correlated. See Supplementary Table S1.

**Figure 1:**
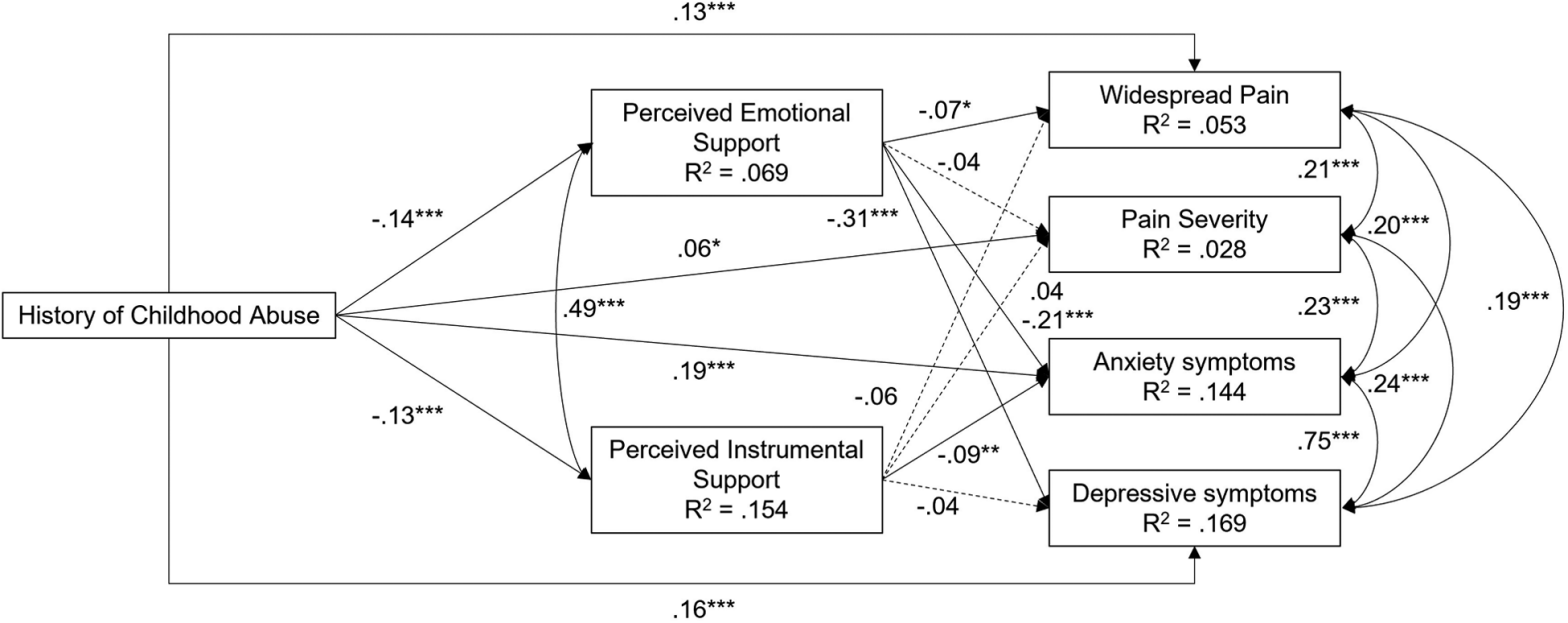
Final mediation model of the effect of history of childhood abuse on pain-related characteristics *via* perceived emotional support and perceived instrumental support (*n* = 1,542). Standardized coefficients are presented. Significance is determined using unstandardized coefficients and robust standard errors. Solid lines indicate significant paths. Dashed lines indicate nonsignificant paths. ‡*p* < .10. *p < .05. **p < .01. ***p < .001. Covariates were included in estimation but are not shown.

As shown in Table 2 and Figure 1, the path model showed significant direct associations from history of childhood abuse to perceived emotional and instrumental support. Approximately 6.9% of the variance in perceived emotional support and 15.4% of the variance in perceived instrumental support were accounted for by the model. Childhood abuse also exhibited significant direct associations with widespread pain, pain severity, anxiety symptoms, and depressive symptoms. Perceived emotional support was significantly associated with lower widespread pain, lower anxiety symptoms, and lower depressive symptoms. Perceived instrumental support was significantly associated with lower anxiety symptoms. Approximately 5.3% of the variance in widespread pain, 2.8% of the variance in pain severity, 14.4% of the variance in anxiety symptoms, and 16.9% of the variance in depressive symptoms were accounted for by the model.

**Table 2:** Unstandardized coefficients, robust SEs, and 95% CIs for final mediation model of the association between history of childhood abuse and pain-related characteristics (*n* = 1,542).

				95% CIs	
	Unstandardized coefficient	Robust SE	*p*	LL	UL
**→Perceived emotional support**					
History of childhood abuse	**−1**.**25**	.**24**	**<**.**001**	**−1**.**728**	**−**.**768**
Age	**−**.**02**	**01**	**<**.**001**	**−**.**032**	**−**.**011**
Female	.31	.18	.088	**−**.046	.664
Married	**1**.**57**	.**20**	**<**.**001**	**1**.**183**	**1**.**957**
**→Perceived instrumental support**					
History of childhood abuse	**−1**.**24**	.**26**	**<**.**001**	**−1**.**745**	**−**.**728**
Age	**−**.001	.01	.903	**−**.012	.011
Female	.10	.19	.584	**−**.269	.477
Married	**2**.**86**	.**21**	**<**.**001**	**2**.**436**	**3**.**274**
**→Widespread pain**					
Perceived emotional support	**−**.**12**	.**05**	.**030**	**−**.**226**	**−**.**012**
Perceived instrumental support	−.09	.05	.104	−.195	.018
History of childhood abuse	**1**.**93**	.**41**	**<**.**001**	**1**.**122**	**2**.**735**
Age	−.01	.01	.485	−.024	.011
Female	**1**.**16**	.**30**	**<**.**001**	.**578**	**1**.**735**
Married	−.34	.35	.319	−1.024	.334
**→Pain severity**					
Perceived emotional support	−.02	.02	.229	−.053	.013
Perceived instrumental support	.02	.02	.221	−.011	.049
History of childhood abuse	.**30**	.**12**	.**012**	.**066**	.**525**
Age	.00	.003	.998	−.007	.007
Female	.**25**	.**10**	.**016**	.**047**	.**452**
Married	**−**.**50**	.**12**	**<**.**001**	**−**.**730**	**−**.**278**
**→Anxiety symptoms**					
Perceived emotional support	**−**.**47**	.**07**	**<**.**001**	**−**.**607**	**−**.**332**
Perceived instrumental support	**−**.**17**	.**06**	.**007**	**−**.**300**	**−**.**047**
History of childhood abuse	**3**.**69**	.**54**	**<**.**001**	**2**.**643**	**4**.**745**
Age	**−**.**04**	.**01**	**<**.**001**	**−**.**067**	**−**.**021**
Female	−.08	.39	.844	−.842	.688
Married	−.64	.43	.138	−1.485	.206
**→Depressive symptoms**					
Perceived emotional support	**−**.**69**	.**07**	**<**.**001**	**−**.**838**	**−**.**544**
Perceived instrumental support	−.07	.07	.288	−.206	.061
History of childhood abuse	**3**.**18**	.**55**	**<**.**001**	**2**.**106**	**4**.**258**
Age	**−**.**03**	.**01**	.**004**	**−**.**053**	**−**.**010**
Female	−.57	.38	.132	−1.309	.172
Married	−.67	.41	.109	−1.478	.148

Boldfaced results are significant at *p *< .05. Intercorrelations among variables at the same level were included but not shown. SE, Standard error; 95% CI, 95% Confidence Interval; LL, lower limit; UL, upper limit.

Total and specific indirect effects are summarized in Table 3. Childhood abuse exhibited a significant total indirect effect on higher widespread pain. Approximately 11.8% of the total effect of childhood abuse on widespread pain was indirect through perceived emotional and instrumental support. This was driven by a significant specific indirect effect through perceived emotional support, which accounted for 54.6% of the total indirect effect. Although the total indirect effect on pain severity was nonsignificant, this was partly due to opposing effects of perceived emotional and instrumental support. Evaluation of the coefficients suggests that 0.6% of the total effect was indirect through perceived emotional and instrumental support. Childhood abuse exhibited a significant total effect on higher anxiety symptoms. Approximately 17.8% of the total effect was indirect through perceived emotional and instrumental support. Perceived emotional support exhibited a higher proportion of the indirect effect (69.1%). Finally, childhood abuse showed a significant total indirect effect on depressive symptoms. Approximately 23.0% of the total effect was indirect through perceived emotional and instrumental support. This was driven by the significant specific indirect effect through perceived emotional support which accounted for 88.1% of the total indirect effect.

**Table 3:** Total and specific indirect effects, bootstrap standard errors, and bias-corrected and accelerated 95% confidence intervals for mediation model of the association between history of childhood abuse and pain-related characteristics (*n* = 1,349).

			BCa bootstrap 95% confidence interval		
	Unstandardized coefficient	Bootstrap SE	LL	UL	Proportion of total indirect effect
**→Widespread pain**					
Total indirect effect	**0** **.** **238**	**0**.**085**	**0**.**098**	**0**.**436**	
→Perceived emotional support	**0**.**130**	**0**.**076**	**0**.**010**	**0**.**312**	54.62%
→Perceived instrumental support	0.108	0.077	−0.023	0.285	45.38%
**→Pain severity**					
Total indirect effect	−0.011	0.023	−0.059	0.034	
→Perceived emotional support	0.030	0.022	−0.007	0.082	42.25%
→Perceived instrumental support	**−0**.**041**	**0**.**024**	**−0**.**098**	**−0**.**003**	57.75%
**→Anxiety symptoms**					
Total indirect effect	**0**.**768**	**0**.**179**	**0**.**445**	**1**.**148**	
→Perceived emotional support	**0**.**531**	**0**.**155**	**0**.**278**	**0**.**876**	69.14%
→Perceived instrumental support	**0**.**237**	**0**.**103**	**0**.**068**	**0**.**485**	30.86%
**→Depressive symptoms**					
Total indirect effect	**0**.**887**	**0**.**213**	**0**.**501**	**1**.**321**	
→Perceived emotional support	**0**.**781**	**0**.**209**	**0**.**421**	**1**.**234**	88.05%
→Perceived instrumental support	0.106	0.098	**−**0.062	0.333	11.95%

Total indirect effect refers to effect of history of childhood abuse through all mediators on specified outcome of interest. BCa, bias-corrected and accelerated; LL, lower limit; UL, upper limit. 95% confidence intervals not containing zero are considered statistically significant and are boldfaced.

## Discussion

Childhood abuse can have long-lasting deleterious effects on social, physical, and psychological health. Investigations into the mechanisms through which childhood abuse impacts health outcomes has seldom considered perceptions of social support. Furthermore, social support is frequently considered as a protective buffer that reduces the impact of adversity on health outcomes. Our study instead explored a potential indirect association, whereby childhood abuse is associated with decrements in perceptions of social support, and more negative perceptions of social support are associated with worse symptoms. The present study provides partial support for our hypotheses and suggests that the association between childhood abuse and sensory and affective pain-related characteristics is partially conveyed through perceptions of social support, particularly emotional support.

### Childhood abuse was associated with poorer perceptions of social support and a more complex pain phenotype

In the present study, patients living with chronic pain who have a history of childhood abuse reported lower perceptions of social support. These findings are in line with previous research. Childhood abuse may mold negative perceptions of others and impact how social information is interpreted (9, 11, 35–37), which may be reflected in less positive perceptions of and less satisfaction with social support (10–12). Childhood abuse may also fuel unhealthy relationship interactions marked by more negativity and less positive behavior that, in turn, results in less support being provided by others (38–40). How accurate these perceptions are and how well they fit the reality of support provision may not be particularly important; previous research suggests that perceptions of support are more important for health-related outcomes than actual support received (41). Additionally, perceptions of social support may be primarily dependent upon observer characteristics (41), including schema and expectations driven by prior experiences like abuse. Similar findings have been reported for the impact of childhood abuse on marital satisfaction in adulthood, which suggest that neither positive nor negative partner characteristics moderated the association (37).

Our findings also suggest that childhood abuse was associated with more widespread pain, higher pain severity, and higher anxiety and depressive symptoms. Indeed, previous research and theory suggests that childhood abuse is associated with poor physical and psychological health outcomes in adulthood (2–5, 42). This extends to a more complex clinical pain phenotype, including more widespread pain, higher pain severity, and higher anxiety and depressive symptoms (4).

### The association between childhood abuse and pain-related characteristics is partially conveyed through perceived emotional support

Although the association between childhood abuse and psychological and physical health outcomes has been well-established in the literature, researchers have highlighted the necessity of understanding the mechanisms of this association. As considered in the current study, perceptions of social relationships may be important to consider. Indeed, social factors (e.g., interpersonal style and social cognition) have been proposed as potential pathways conveying the impact of childhood abuse on health outcomes (8). Yet, other pathways are important as well, including the influence of childhood abuse on health risk and promotive behavior and posttraumatic stress symptoms (8). Thus, it is unsurprising that significant, direct associations were found and maintained even in the present mediation model.

Our findings, however, suggest that the association between childhood abuse and sensory and affective pain-related characteristics, including widespread pain, anxiety symptoms, and depressive symptoms, could be partially explained by perceptions of social support. Similar to our findings, previous research has found that perceptions of social support and perceived relationship quality were important mechanisms linking childhood abuse with depressive symptoms (27), allostatic load in middle adulthood (12), as well as worse health perceptions, worse mental health, and more somatic complaints (13). Furthermore, recent research found that marital quality, composed of perceptions of support, strain, and disagreement, conveyed the association between childhood abuse and negative affect among older adults (43). Thus, perceptions of social support are a potentially modifiable mechanism that should be further investigated. Of note, although variance in widespread pain was better accounted for by the model than variance in pain severity, both outcomes exhibited low variance explained suggesting that other predictors are important to consider for sensory pain-related characteristics. Instead, the present model appears to better predict affective pain-related characteristics.

The association between childhood abuse and widespread pain is particularly interesting. Compared to pain severity, widespread pain may be more characteristic of nociplastic pain, in which central nervous system factors are believed to play a part in the spread or amplification of pain (25, 44). Stressors have been implicated as precipitating factors of nociplastic pain (25). Thus, low satisfaction with social support, negative social perceptions, and accompanying relationship distress may be sources of chronic stress that contribute to poor physical health including nociplastic pain (8). Alternatively, survivors of childhood abuse may not benefit from favorable perceptions of social support, which are shown to reduce perceptions of stress, improve coping strategies, and reduce the physiological stress response to pain (17).

The lack of findings with pain severity may also be associated with the time frame in which it is evaluated. The measure of widespread pain is indicative of chronicity, whereas the measure of pain severity considered the preceding week. Our model likely reflects long processes in which childhood abuse negatively impacts social relationships across the lifespan, and dissatisfying social perceptions and relationships influence perceptions of stress as well as present direct sources of distress that compound over time (45). On the other hand, pain severity may be a brief snapshot of sensory experiences. Future research should consider dynamic trajectories of pain severity and whether these instead are associated with social perceptions.

Additionally, instrumental support was associated with higher pain severity in the model, although this direct association was nonsignificant. This likely contributed to the significant indirect effect linking childhood abuse to lower pain severity through instrumental support. The model findings suggest that instrumental support may function as a solicitous form of social support that promotes and reinforces pain behavior like expressions of severity (46). Thus, the availability of instrumental support may encourage individuals to acknowledge the severity of their pain in order to receive much needed assistance with daily tasks.

The findings also suggest that, overall, perceived emotional support better predicts sensory and affective pain-related characteristics compared to perceived instrumental support. The benefit of each form of support may depend on what the individual requires in specific situations (14). Emotional support may be more beneficial for chronic health conditions (47), whereas instrumental support may be more valuable for specific needs.

### Strengths and limitations

The present study had many strengths. We evaluated these associations in a large sample of patients with pain using measures of both emotional and instrumental social support, and also broadly conceptualized pain-related characteristics as encompassing both sensory and affective components. Numerous limitations should also be acknowledged. We tested a mediation model which imposed a specific directional effect and our results reflect our statistical approach. However, the data were cross-sectional which limits our ability to make any causal claims. There are likely bidirectional relationships between social support perceptions and pain-related characteristics. Our goal, however, was not to propose a specific direction of effects to the exclusion of alternative models, but to evaluate a model that may have implications for chronic pain interventions. Our measure of abuse was limited due to the data being collected in the context of clinical care and the necessity to keep measures brief to ease participant burden. Type and timing of abuse should be investigated in future research. Additionally, many victims of abuse do not acknowledge or label the experience as abuse. Measures that provide behavioral descriptions of what the abuse would entail would likely result in higher prevalence rates. The participants also had various pain diagnoses and mixed etiology. This may contribute to important, yet indistinguishable, heterogeneity in the current sample. Future research should consider these factors. Additionally, due to the secondary nature of the present analyses, we did not have measures related to other forms of social integration or negative social perceptions such as hostility and perceived rejection. Future research should consider the role of social integration, which may be more likely to exhibit direct effects on health outcomes (14), as well as the impact of negative social perceptions on health outcomes, which may be more strongly associated with poor health outcomes compared to conceptually positive experiences of emotional and instrumental support (48).

### Clinical implications and conclusion

Despite these limitations, understanding the impact of childhood abuse on perceptions of social support and, in turn, social support on pain-related characteristics is important to understand as chronic pain management techniques and interventions that promote the value of social support (e.g., support groups, engaging spouses or loved ones in care) are often recommended. Yet, patients with a history of childhood abuse victimization may *perceive* support differently which would have important implications for these pain management techniques and interventions. Thus, it is critical for healthcare providers to consider the interpersonal history of patients. Previous studies have demonstrated the importance of screening all chronic pain patients for a history of abuse (49, 50). Doing so can help guide intervention administration and modifications. Indeed, patients with a history of childhood abuse may especially benefit from interventions that improve the sense of safety and care in their relationships.

The findings also extend to the patient-provider relationship. Patients' trust in the recommendations of their provider is predicated on trust in the relationship they have with that provider. There is evidence that childhood abuse results in patients having less trust in their providers, as well as providers rating these patients as more difficult (50–53). This bidirectional challenge can undermine the very safety and trust needed to be able to bring up and discuss the role that childhood abuse could have or be having on the overall management of their pain, thus making long term success and improvement even harder to achieve. Further research into the impact of trauma-informed care and abuse screening in the setting of chronic pain management is needed to explore ways to make the seeking of care safe and supportive.

Survivors of childhood abuse exhibit a worse clinical pain phenotype, highlighting the critical need to understand the various mechanisms driving this association. Researchers and clinicians should consider the role of social perceptions within this complex relationship, including their impact on the patient-provider relationship. Interventions that improve patients' sense that others love and care for them may contribute to symptom improvement.

## Data Availability

The raw data supporting the conclusions of this article will be made available by the authors, without undue reservation.
