# Supplementary material for: Perceived social support partially mediates the association between childhood abuse and pain-related characteristics
Source: Front Pain Res (Lausanne). 2022 Dec 22;3:1075605. doi: 10.3389/fpain.2022.1075605 (PMC9815443; doi:10.3389/fpain.2022.1075605)
Supplement: Supplementary file 1 [file Table1.docx]

| Supplemental Table 1 | |  |  |  |  |  |  |  |  |  |
| --- | --- | --- | --- | --- | --- | --- | --- | --- | --- | --- |
| Bivariate Correlations for Study Variables | | |  |  |  |  |  |  |  |  |
|  |  | 1 | 2 | 3 | 4 | 5 | 6 | 7 | 8 | 9 |
| 1 | History of childhood abuse | -- |  |  |  |  |  |  |  |  |
| 2 | Age | **-0.10** | -- |  |  |  |  |  |  |  |
| 3 | Female | **0.14** | **-0.09** | -- |  |  |  |  |  |  |
| 4 | Married | **-0.12** | **0.22** | **-0.11** | -- |  |  |  |  |  |
| 5 | Perceived emotional support | **-0.14** | -0.03 | 0.00 | **0.24** | -- |  |  |  |  |
| 6 | Perceived instrumental support | **-0.17** | **0.09** | -0.04 | **0.38** | **0.54** | -- |  |  |  |
| 7 | Widespread pain | **0.17** | -0.05 | **0.12** | **-0.09** | **-0.12** | **-0.13** | -- |  |  |
| 8 | Pain severity | **0.08** | -0.04 | **0.08** | **-0.14** | -0.05 | -0.03 | **0.24** | -- |  |
| 9 | Anxiety symptoms | **0.24** | **-0.12** | 0.03 | **-0.18** | **-0.29** | **-0.26** | **0.24** | **0.24** | -- |
| 10 | Depressive symptoms | **0.21** | **-0.08** | -0.01 | **-0.18** | **-0.37** | **-0.26** | **0.23** | **0.25** | **0.79** |
| *Note.* Boldfaced results are significant at *p* < .05. | | | | | | | | | | |
